# Supplementary material for: The MEK/ERK/miR-21 Signaling Is Critical in Osimertinib Resistance in EGFR-Mutant Non-Small Cell Lung Cancer Cells
Source: Cancers (Basel). 2021 Nov 29;13(23):6005. doi: 10.3390/cancers13236005 (PMC8657072; doi:10.3390/cancers13236005)

SUPPLEMENTARY INFORMATION

The MEK/ERK/miR-21 signaling is critical in Osimertinib resistance in EGFR-mutant non-small cell lung cancer cells

Wen-Chien Huang[1,2], Vijesh Kumar Yadav[3], Wei-Hong Cheng[4], Chun-Hua Wang[5,6], Ming-Shou Hsieh [3], Ting-Yi Huang[3], Shiou-Fu Lin[7], Chi-Tai Yeh[3,4,8], Kuang-Tai Kuo[9,10]*

1 Department of Medicine, MacKay Medical College, New Taipei City, 252, Taiwan.

2 Division of Thoracic Surgery, Department of Surgery, MacKay Memorial Hospital, Taipei, 104, Taiwan.

3 Department of Medical Research & Education, Taipei Medical University - Shuang Ho Hospital New Taipei City, 235, Taiwan.

4 Division of Hematology and Oncology, Department of Internal Medicine, Taipei Medical University-Shuang Ho Hospital, New Taipei City 235, Taiwan.

5 Department of Dermatology, Taipei Tzu Chi Hospital, Buddhist Tzu Chi Medical Foundation, New Taipei City, 231, Taiwan.

6 School of Medicine, Buddhist Tzu Chi University, Hualien, 970, Taiwan.

7 Department of Pathology, Taipei Medical University - Shuang Ho Hospital, New Taipei City, 235, Taiwan.

8 Department of Medical Laboratory Science and Biotechnology, Yuanpei University of Medical Technology, Hsinchu 300, Taiwan.

9 Division of Thoracic Surgery, Department of Surgery, School of Medicine, College of Medicine, Taipei Medical University, Taipei, 110, Taiwan.

10 Division of Thoracic Surgery, Department of Surgery, Taipei Medical University - Shuang Ho Hospital, New Taipei City, 235, Taiwan.

* Correspondence: Email: ktkuo@tmu.edu.tw; Tel.: +886-2-2490088 ext. 8119

Supplementary Table S1. Clinicopathological characteristics of NSCLC patients (N=20)

**Parameters**	**N**	**%**
Age (years)	56.4 ± 2.8	
Gender (male)	20	100%
Smoke (n/%)	16	75%
Histology		
Adenocarcinoma (n/%)	16	60%
Squamous cell carcinoma (n/%)	4	40%
Pathological grade		
Poorly differentiated (n/%)	16	80%
Moderately differentiated (n/%)	3	15%
Well-differentiated (n/%)	1	5%
Tumor size		
＞5 cm (n/%)	10	50%
＜=5 cm (n/%)	10	50%
Osimertinib responder		
Yes (n/%)	10	50%
No (n/%)	10	50%

Supplementary Table S2. qPCR primers

Gene name	Forward sequence	Reverse sequence
FAP	ATGAGCTTCCTCGTCCAATTCA	AGACCACCAGAGAGCATATTTTG
ERK	ATGTCATCGGCATCCGAGAC	GGATCTGGTAGAGGAAGTAGCA
STAT3	CAGCAGCTTGACACACGGTA	AAACACCAAAGTGGCATGTGA
VIM	GACGCCATCAACACCGAGTT	CTTTGTCGTTGGTTAGCTGGT
A-SMA	AAAAGACAGCTACGTGGGTGA	GCCATGTTCTATCGGGTACTTC
E-cad	CGAGAGCTACACGTTCACGG	GGGTGTCGAGGGAAAAATAGG
β-actin	CTGTCTGGCGGCACCACCAT	GCAACTAAGTCATAGTCCGC

Supplementary Figure S1 The representative examples of Osimertinib-resistant and Osimertinib-sensitive cell lines deriving from patients (computed tomography).

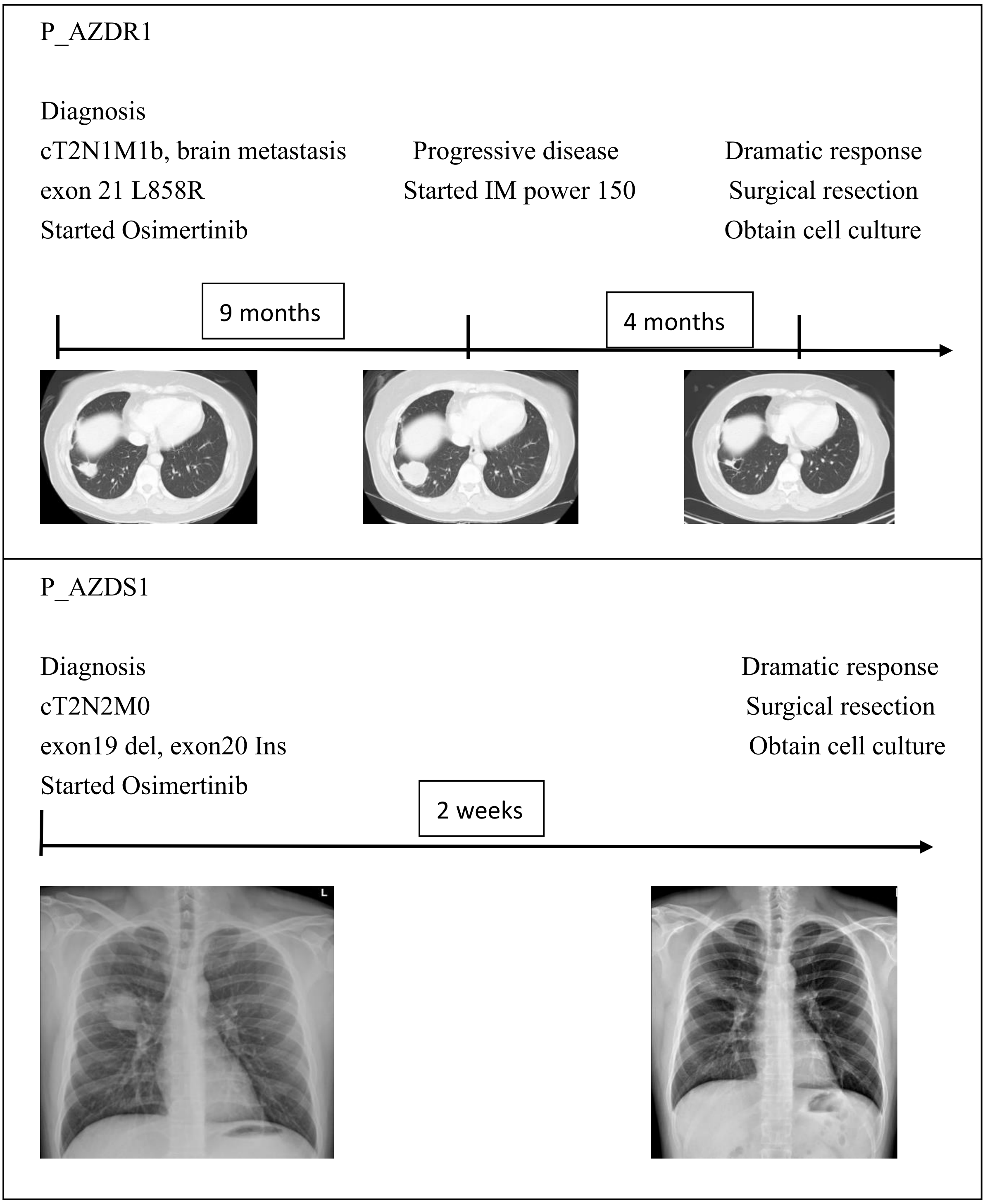

Supplementary Figure S2 Primary cancer cell isolation process.

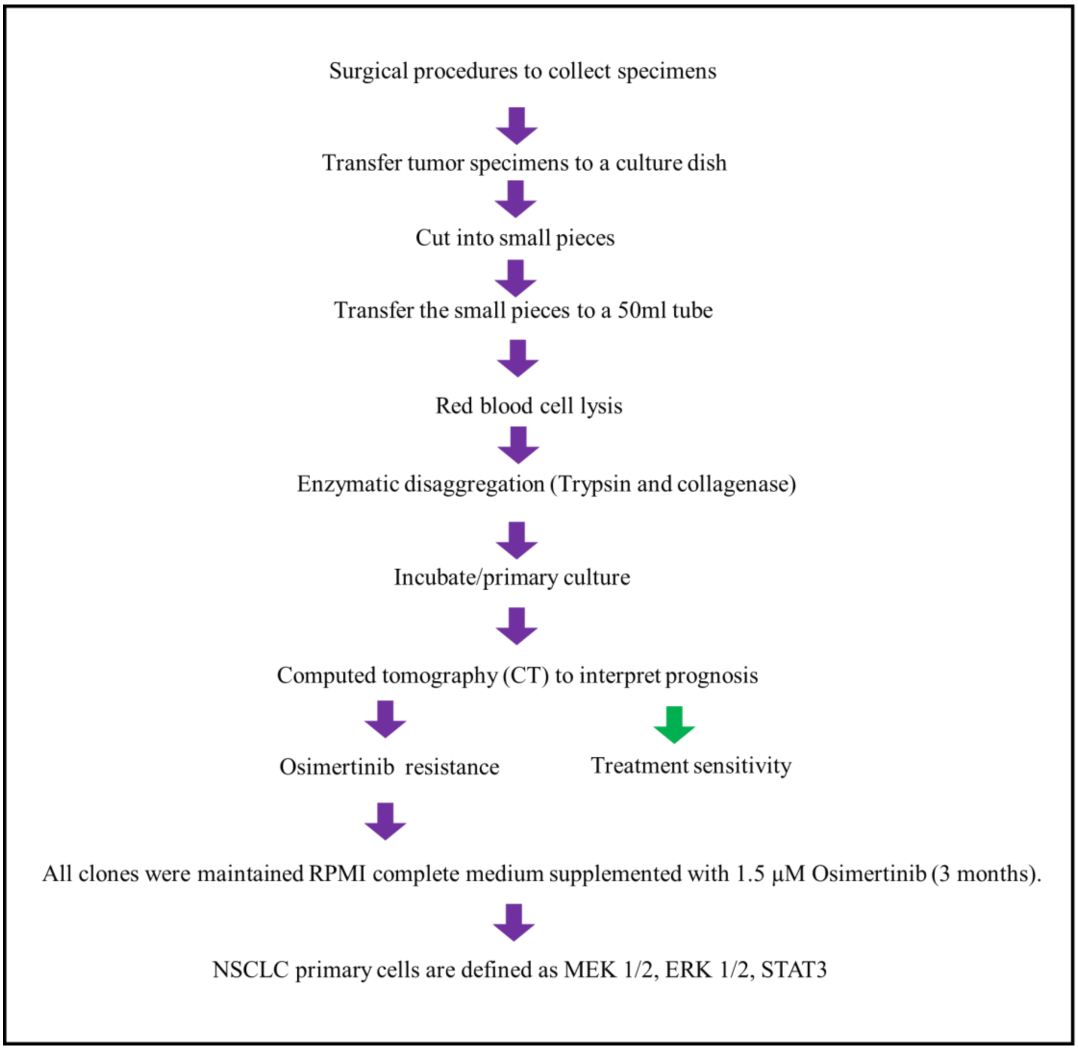

Supplementary Figure S3 Inhibiting mir-21-5p does the job in commercial cell lines (PC9 and A549).

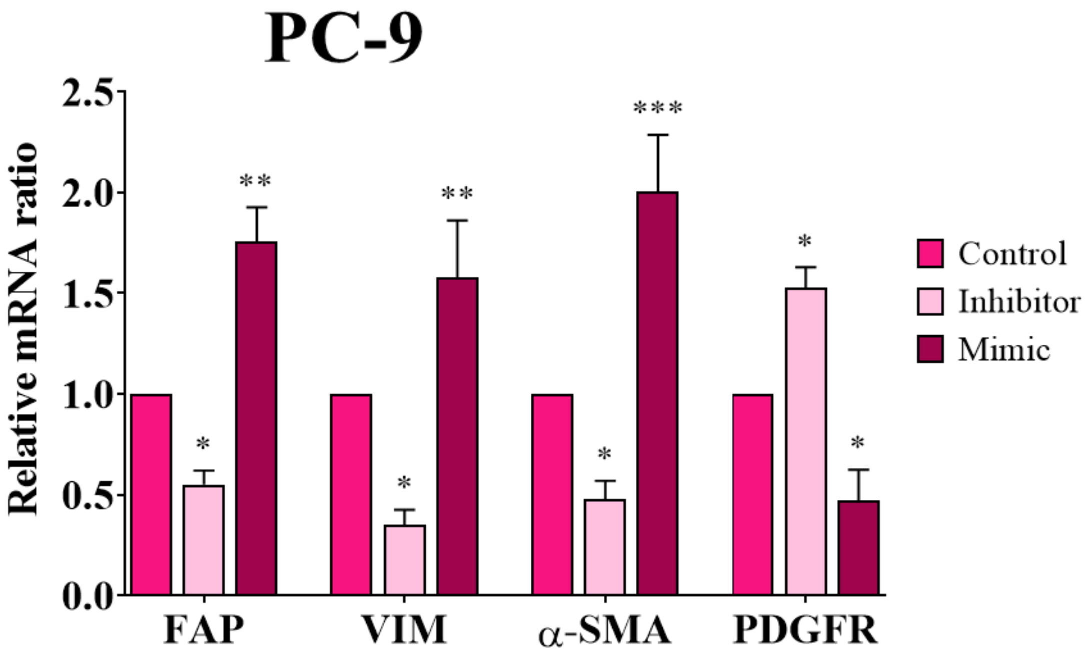

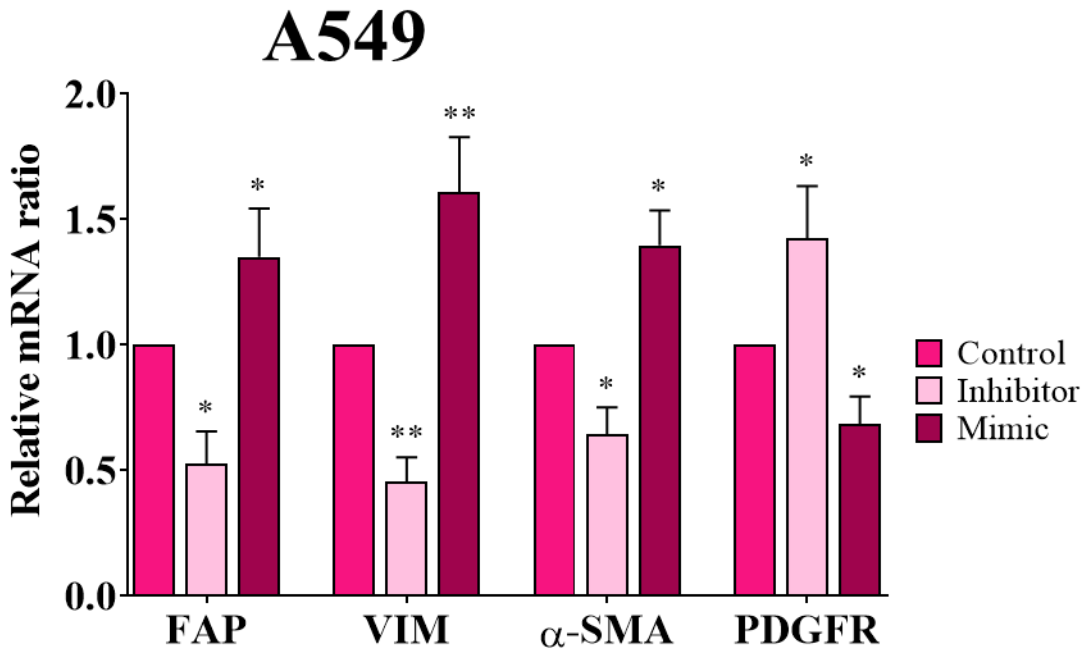

Supplementary Figure S4. Full-size blots of Figure 2F

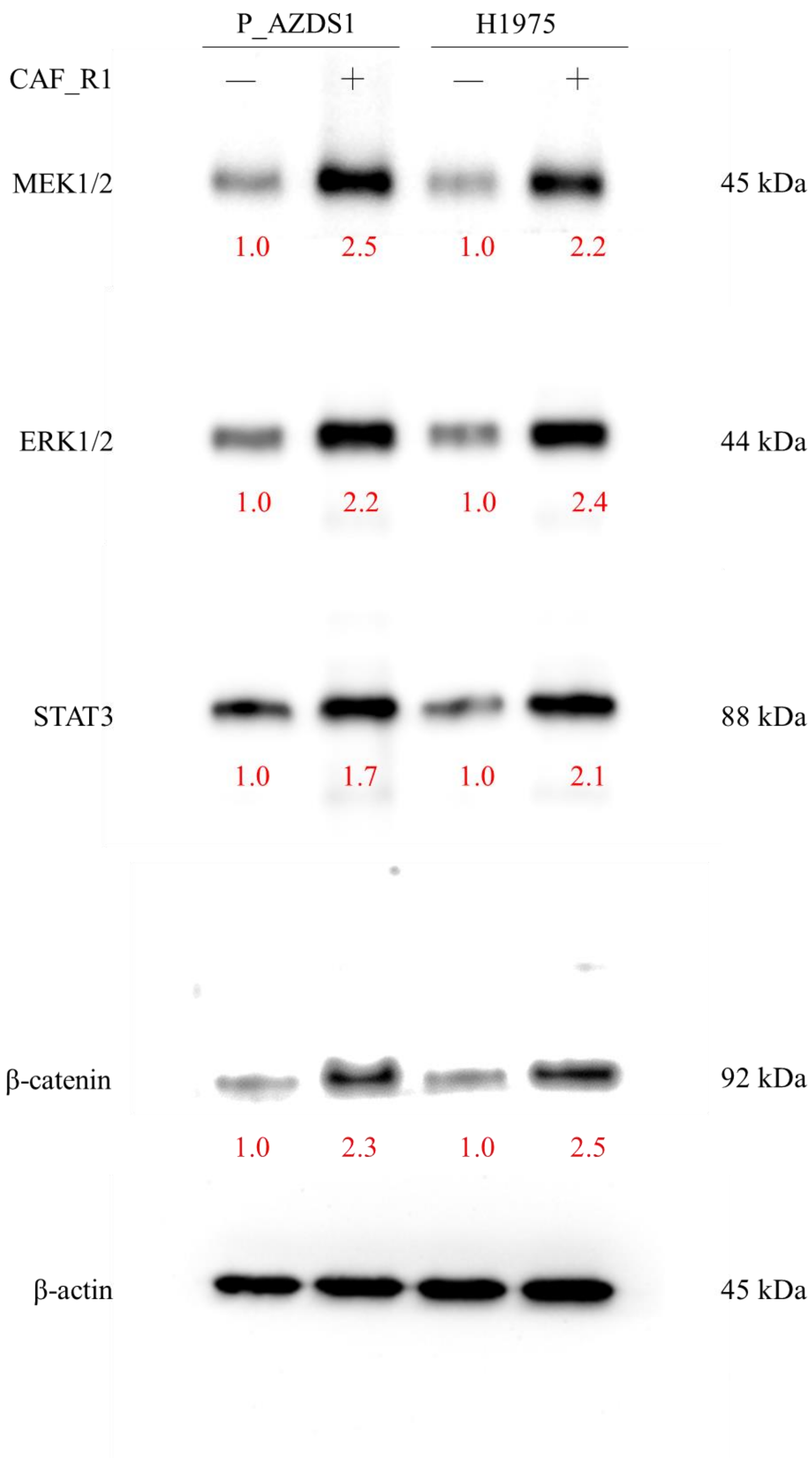

Supplementary Figure S5. Full-size blots of Figure 3F

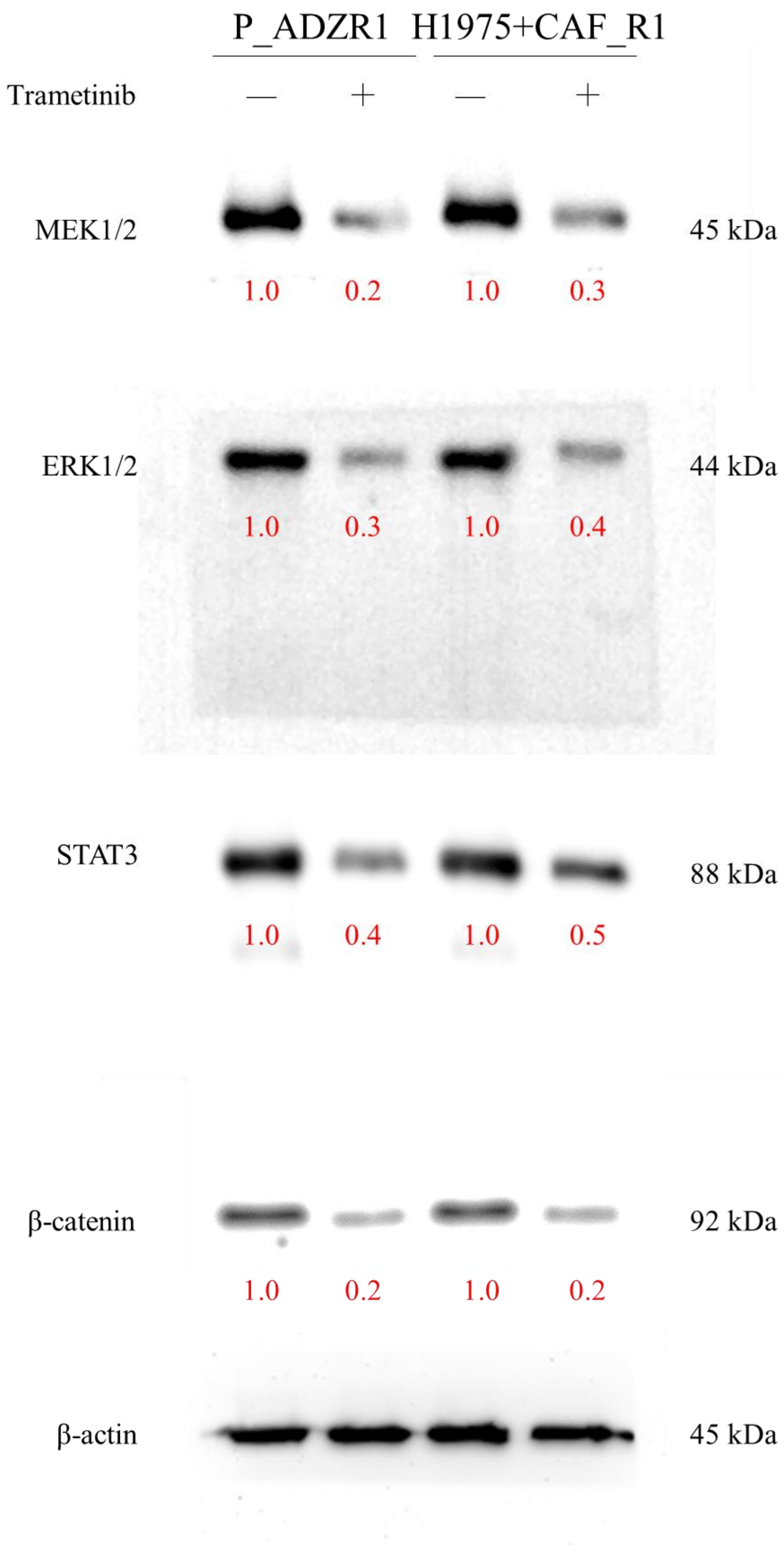

Supplementary Figure S6. Full-size blots of Figure 4C.

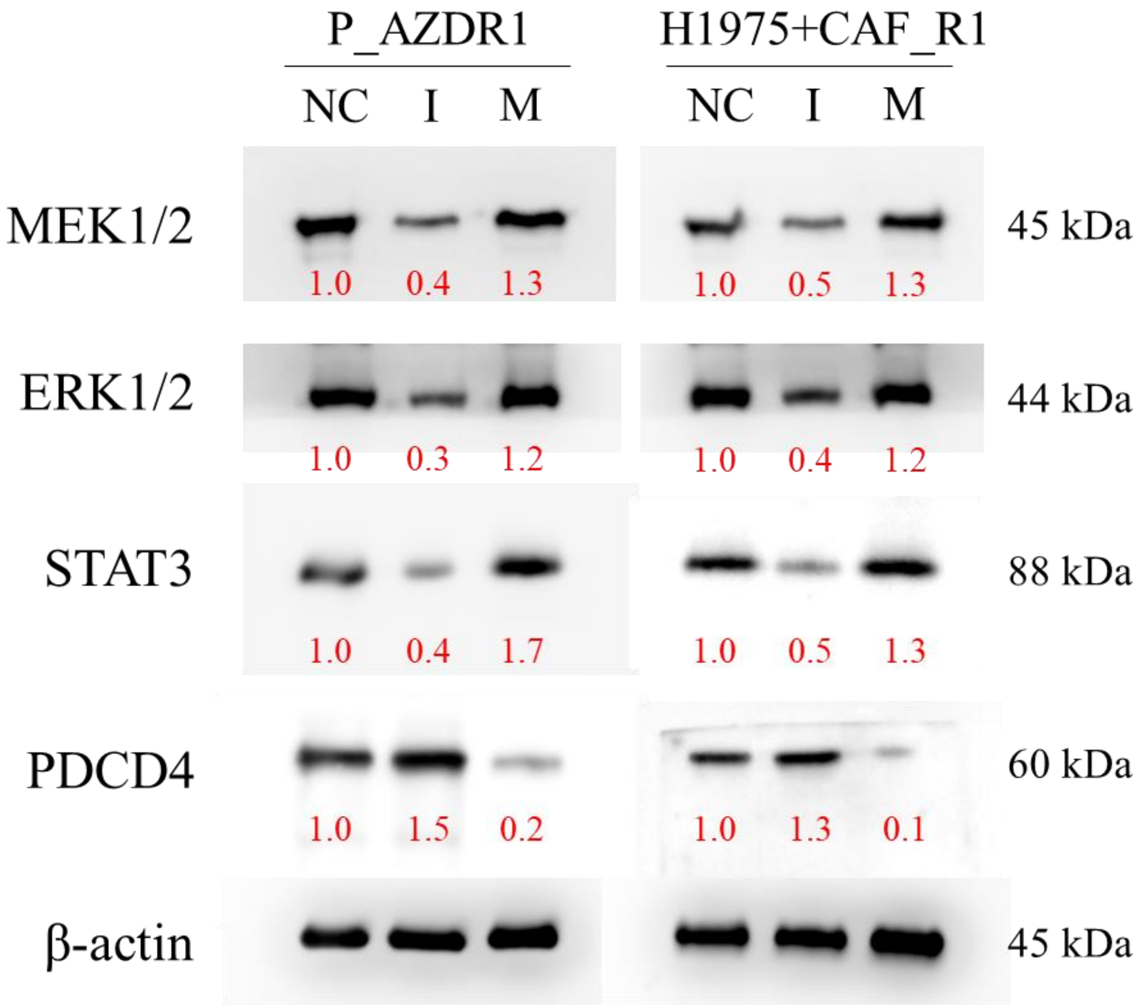

Supplement: Supplementary file 1 [file cancers-13-06005-s001.zip › cancers-1424551-supplementary.pdf]
